# Robust Segmentation of the Full Cerebral Vasculature in 4D CT of Suspected Stroke Patients

**DOI:** 10.1038/s41598-017-15617-w

**Published:** 2017-11-15

**Authors:** Midas Meijs, Ajay Patel, Sil C. van de Leemput, Mathias Prokop, Ewoud J. van Dijk, Frank-Erik de Leeuw, Frederick J. A. Meijer, Bram van Ginneken, Rashindra Manniesing

**Affiliations:** 1 Department of Radiology and Nuclear Medicine, Radboud University Medical Center, Geert Grooteplein 10, 6525GA Nijmegen, The Netherlands; 2 Department of Neurology, Radboud University Medical Center, Geert Grooteplein 10, 6525GA Nijmegen, The Netherlands

## Abstract

A robust method is presented for the segmentation of the full cerebral vasculature in 4-dimensional (4D) computed tomography (CT). The method consists of candidate vessel selection, feature extraction, random forest classification and postprocessing. Image features include among others the weighted temporal variance image and parameters, including entropy, of an intensity histogram in a local region at different scales. These histogram parameters revealed to be a strong feature in the detection of vessels regardless of shape and size. The method was trained and tested on a large database of 264 patients with suspicion of acute ischemia who underwent 4D CT in our hospital in the period January 2014 to December 2015. Five subvolumes representing different regions of the cerebral vasculature were annotated in each image in the training set by medical assistants. The evaluation was done on 242 patients. A total of 16 (<8%) patients showed severe under or over segmentation and were reported as failures. One out of five subvolumes was randomly annotated in 159 patients and was used for quantitative evaluation. Quantitative evaluation showed a Dice coefficient of 0.91 ± 0.07 and a modified Hausdorff distance of 0.23 ± 0.22 mm. Therefore, robust vessel segmentation in 4D CT is feasible with good accuracy.

## Introduction

Vessel segmentation in medical imaging is the cornerstone of many applications. For example, in acute ischemic stroke which is caused by a disturbance of blood supply to the brain, segmentation of the cerebral vasculature is important for improved visualization and for the detection and quantification of vessel occlusions, stenoses and for the evaluation of collateral flow. Vascular occlusions in the proximal segments of the intracranial arteries are targets for endovascular thrombectomy, that is, the mechanical removal of the clot which has been shown to improve patient outcome when performed within, or possibly even beyond, 6 hours after onset of symptoms^1^. Other applications include detection and characterization of vascular malformations or aneurysms and local arterial input function selection for perfusion analysis.

The task of vessel segmentation has led to a vast amount of literature in the past decades^2,3^. Related work on cerebral vasculature has focused solely on the accuracy of finding the vessel boundaries; none have demonstrated that their proposed method works on large number of patients that are seen in everyday clinical practice. In Table 1, an overview is shown of related work on cerebral vessel segmentation.

**Table 1:** Overview of related work on cerebral vessel segmentation and vessel challenges, grouped per modality. The number of patients indicate the number of patients used in the quantitative evaluation. ^†^Used pattern recognition in their methods.

Author	Method	Modality	N	Anatomy	Reference
Flasque *et al*.^4^	Centerline modeling and tracking	MRA	0	full brain	n/a
Passat *et al*.^30^	Atlas registration and hit-or-miss transform	MRA	50	full brain	Passat *et al*.^31^
Hassouna *et al*.^32^	3D markov rand. field and max. pseudo likelihood est.	TOF-MRA	1	full brain	wooden phantom
Yan *et al*.^33^	Capillary geodesic active contour	MRA	0	CoW	Lorigo *et al*.^34^
Law *et al*.^35^	Variance-based edge detection	TOF-MRA	0	CoW	n/a
Gao *et al*.^36^	Expectation maximization modeling and curve evaluation	TOF-MRA	10	CoW	manual
Wang *et al*.^37^	Otsu’s threshold and Gumbel distribution based threshold	TOF-MRA	10	CoW	manual
Robben *et al*.^11^^,†^	Cascade classifier and directed graph evaluation	TOF-MRA	50	CoW	Bogunovic *et al*.^38^
Manniesing *et al*.^39^	Bone masking and levelset	CTA	10	CoW	manual
Schaap *et al*.^40^	Bayesian tracking of tubular model	CTA	28	ICA	manual
Hernandez *et al*.^41^	Non-parametric geodesic active regions	CTA, 3DRA	10	CoW	manual
Schaap *et al*.^18^	Coronary artery centerline extraction (challenge)	CTA	24	coronaries	manual
Hameeteman *et al*.^19^	Carotid bifurcation detection (challenge)	CTA	41	ICA	manual
Kirişli *et al*.^20^	Coronary artery stenosis detection (challenge)	CTA	30	coronaries	manual
Kiros *et al*.^12^^,†^	Multiscale dictionary learning (challenge participant)	CT	20	lung	VESSELS12
Mendrik *et al*.^10^	Global threshold on area under intensity curve	4D CT	20	slabs on CoW	manual
Our method^†^	Random forest classifier with local histogram features	4D CT	159	full brain	manual

**Figure 1:**
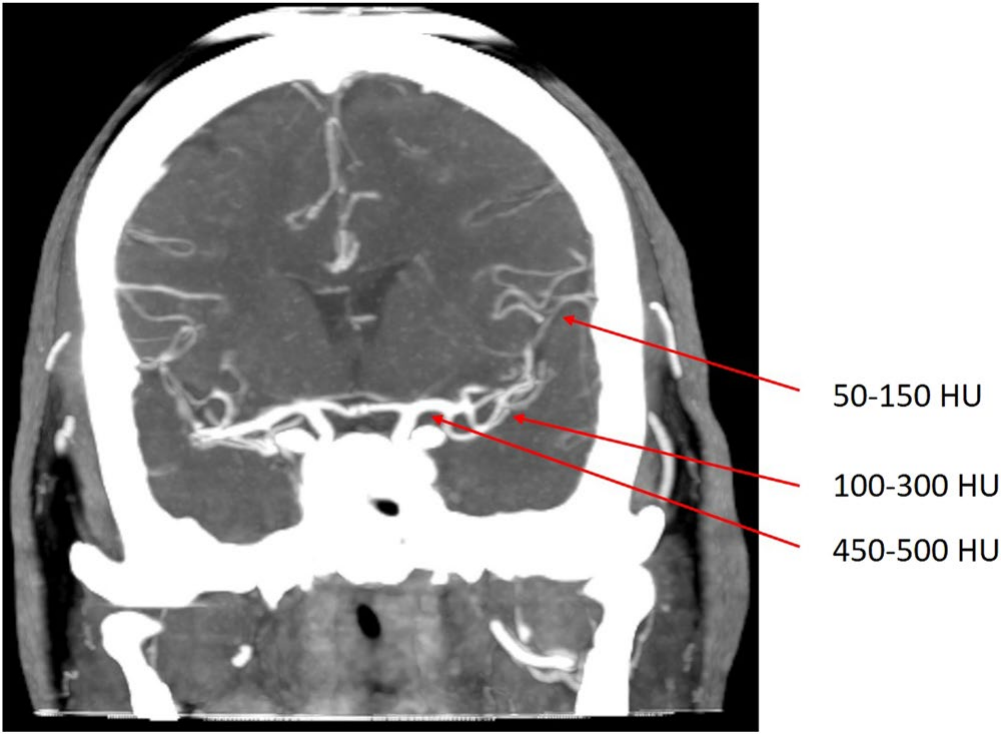
Coronal view of a temporal maximum intensity projection visualizing part of the middle cerebral artery including the M1, M2 and M3 segments. Intensity differences from proximal to distal in a nonaffected vessel can reach up to 450 HU and higher. Vessel occlusions, vessel wall calcifications, collateral flow, clip and stent artifacts have a large influence on the continuity of intensity values along the vessel.

**Figure 2:**
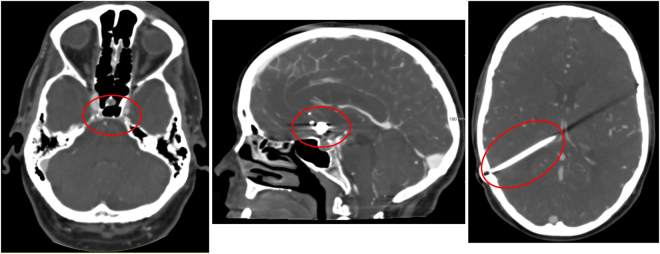
Examples of difficulties encountered in vessel segmentation. From left to right: Skull base region, arteries and veins surrounded by hyperdens bony structures in their course through the skull base, which renders difficulties in separating them from each other; patient with coils placed at the anterior communicating artery; patient with ventricular shunt causing a linear artifact in the left cerebral hemisphere.

Most methods use centerline tracking, active contours or a combination of both^4^, with parameters set at a global level to represent both large and small vessels. Tracking based approaches will have difficulty achieving the desired level of robustness on full brain vessel segmentation. These methods require some form of initialization, as intensity values vary along the vessel. In CT Angiography (CTA) intensity values naturally drop to the values of background tissue, which is around 20 to 40 HU for cerebral white and grey matter, at that point marked as partial volume voxels. However, in the proximal intracranial arteries, the internal carotid artery (ICA) and vertebral artery, intensity values of 200 to 500 HU and higher can be measured (Fig. 1). In addition, nonnatural variations caused by pathology, such as vessel occlusions and arteriovenous malformations, or imaging artifacts, resulting from the presence of clips or stents, have a major influence on the continuity of the intensity along the vessels (Fig. 2). Therefore, this may even pose a challenge for tracking based methods with adaptive properties.

Another problem encountered in segmentation of the cerebral vasculature is the area of the skull base. In this region the ICA, the jugular bulb and soft bone tissue are close to each other with similar intensities between these structures. Vessel pathologies (e.g. calcifications and aneurysms) often appear in the skull region compared to other parts in the brain^5^. Region based approaches that find the vessel boundaries directly without first centerline tracking^6^, may have difficulties in this area as well.

CT is the primary imaging modality in an emergency setting because of its short acquisition time and because of the fewer contraindications compared to magnetic resonance (MR). Furthermore, CT has a high diagnostic value in the acute phase of stroke, due to its high sensitivity to hemorrhages and vascular pathology^7–9^. Modern CT scanners are capable of acquiring contrast-enhanced 4D CT images with high spatial and temporal resolution at large coverage, enabling visualization of the contrast dynamics at submillimeter resolution of the full brain. A 4D CT acquisition is less critical concerning timing of the peak arterial enhancement compared to conventional 3D CTA.

In this work we consider vessel segmentation in 4D CT. Only one related vessel segmentation method was found in 4D CT^10^, which was based on fixed thresholding of a global intensity histogram. Another approach towards vessel segmentation is a method based on pattern recognition. This way local differences of vessel shape and intensity can be learned, which makes it less prone to discontinuities of the vessels e.g. due to the presence of pathologies or foreign objects. In addition, a pattern recognition method requires no explicit seed points for initialization, as opposed to tracking based methods.

Pattern recognition based approaches have hardly been used for vessel segmentation. One reason may be the difficulty of establishing a reliable reference standard. A precise manual delineation of the tortuous small vessels is difficult and time-consuming. Two related works have been found based on pattern recognition: on vessel segmentation in MR angiography (MRA)^11^ and on vessel segmentation in lung CT^12^. The latter achieved a top rank in the VESSEL12 challenge^13^ demonstrating the potential of such an approach.

In this work, we present a pattern recognition approach for segmentation of the complete cerebral vasculature in 4D CT. The proposed method emphasizes robustness and should be capable of handling patient data typically seen in everyday clinical practice. The method also emphasizes completeness over accuracy in defining the vessel boundaries by including the segmentation of the smaller vessels more distally in the vascular tree. Obtaining accurate boundary segmentations is easier starting from the complete cerebral vasculature than the other way around. Furthermore, completeness of the segmentation is more important, because the desired accuracy of the lumen boundary is dependent on the subsequent application.

## Results

### Observer Variability

**Figure 3:**
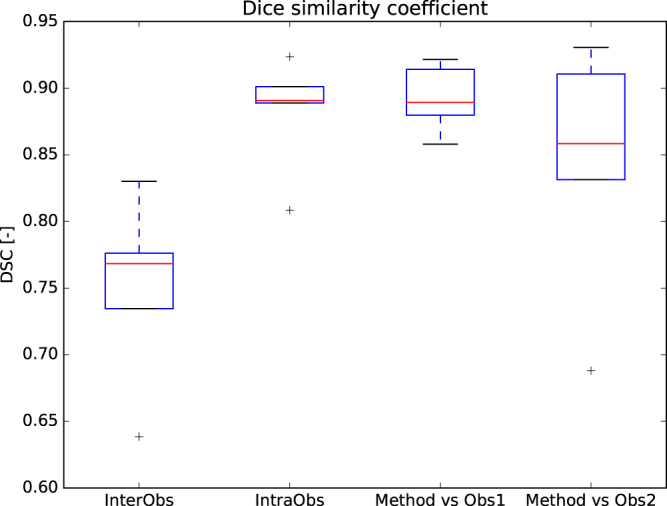
Boxplot of the Dice similarity coefficient calculated for the inter-observer variability, intra-observer variability and the proposed method versus each observer independently.

Table 2 shows the quantitative evaluation of the inter-observer variability, intra-observer variability and the performance of the method in comparison to each observer for a subset of five 4D CT images. DSC is Dice similarity coefficient^14^, HD is Hausdorff distance^15^, MHD is modified HD^16^, 95% HD is 95^th^ percentile HD, CMD is contour mean distance^17^ and AVD is absolute volume difference. The DSC for the inter-observer variability was 0.75 ± 0.06. The method scored higher, although this difference is only significant in observer 1. A boxplot graphical overview is shown in Fig. 3 where the center line indicates the median value, the box edges depict the 25^th^ and 75^th^ percentiles. The whiskers show the extremes at 1.5 inter-quartile range excluding the outliers, indicated as +.

**Table 2:** Quantitative evaluation of inter-observer variability, intra-observer variability and method versus each observer independently (n = 5), reported as mean ± standard deviation. *p*-values were computed with a paired sample *t*-test between the inter-observer variability and proposed method for each evaluation measure. ^†^Indicates a significant difference (*p*-value < 0.05, i.e. less than 5% chance that the samples have identical averages).

	Inter-observer (n = 5)	Intra-observer (n = 5)	Method vs. observer 1 (n = 5)	Method vs. observer 2 (n = 5)
DSC	0.75 ± 0.06	0.88 ± 0.04	0.89 ± 0.02^†^	0.84 ± 0.09
HD (mm)	17.98 ± 2.01	11.72 ± 2.03	20.34 ± 2.35	16.21 ± 3.80
MHD (mm)	0.61 ± 0.10	0.15 ± 0.03	0.76 ± 0.12	0.28 ± 0.11
95% HD (mm)	7.57 ± 3.07	0.86 ± 0.43	9.41 ± 2.22	5.59 ± 3.45
CMD (mm)	0.78 ± 0.14	0.20 ± 0.03	0.85 ± 0.13	0.35 ± 0.13
AVD (%)	44.49 ± 18.46	4.31 ± 3.50	14.49 ± 3.50^†^	24.16 ± 26.66

### Qualitative Evaluation

**Figure 4:**
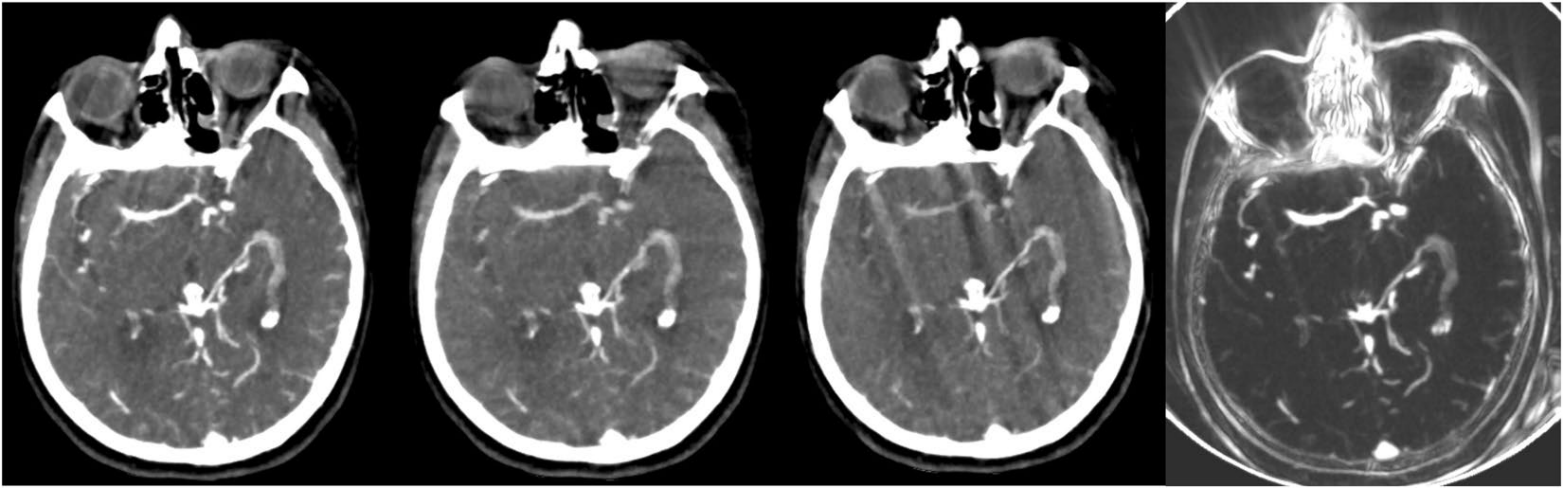
Axial view of three consecutive temporal acquisitions in a patient with beam hardening and motion artifacts in a single 4D CT acquisition and the resulting Weighted Temporal Variance (WTV) image (right).

Visual inspection of all segmentations (n = 242) revealed a total of 16 failures (<8%). In 8 patients this failure was the result of severe motion artifacts in multiple volume acquisitions unaccounted for by the registration of the 4D CT or due to beam hardening artifacts during the acquisition. This resulted in a high intensity variation over time leading to erroneous segmentation. An example of this is shown in Fig. 4. These 16 failures were not considered for quantitative evaluation. Smaller movement artifacts outside the cranial cavity (e.g. eye or jaw movement) posed no problem for the method.

**Figure 5:**
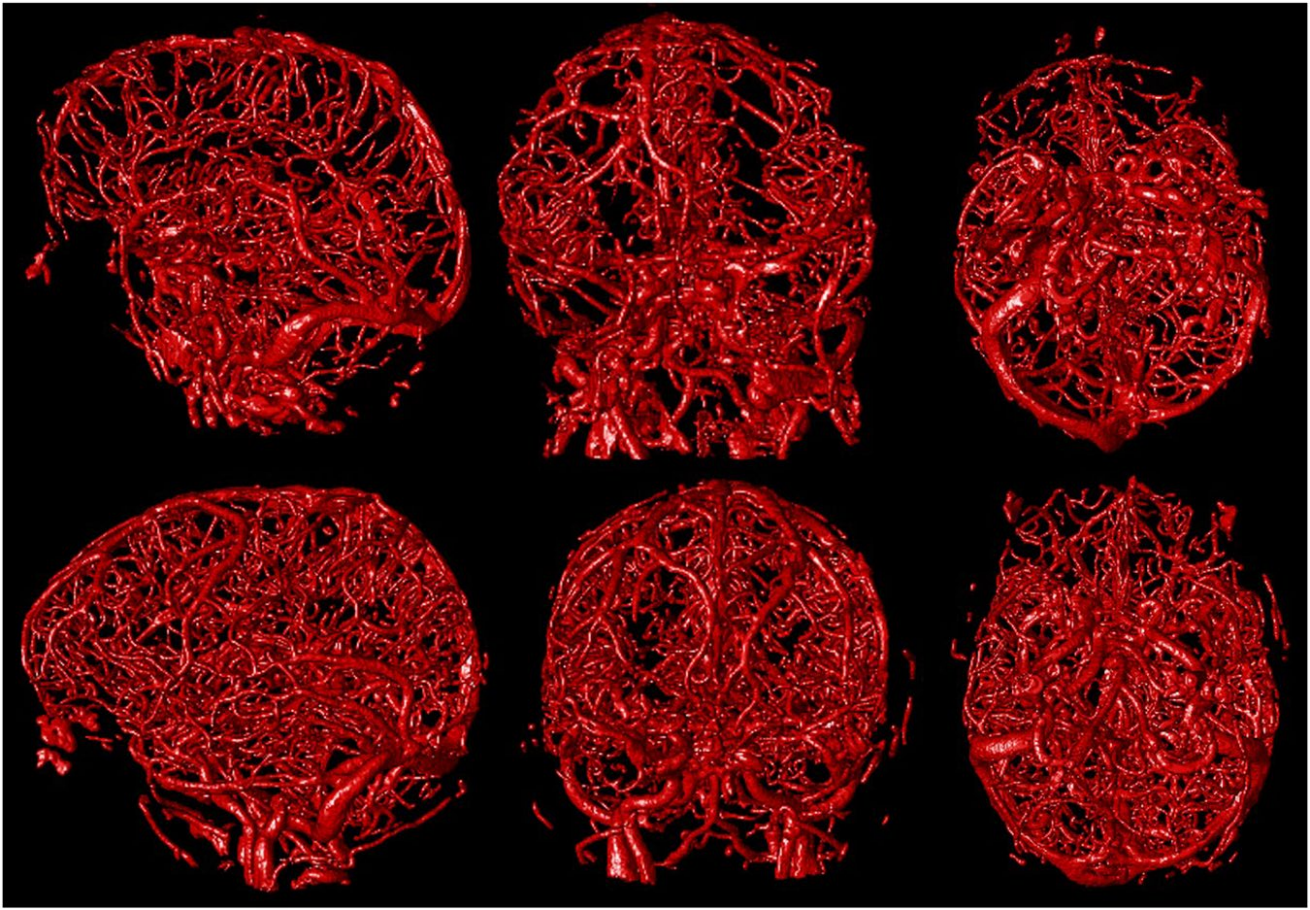
Left to right sagittal, coronal and axial view of a 3D rendering of the segmentation of two different patients.

**Figure 6:**
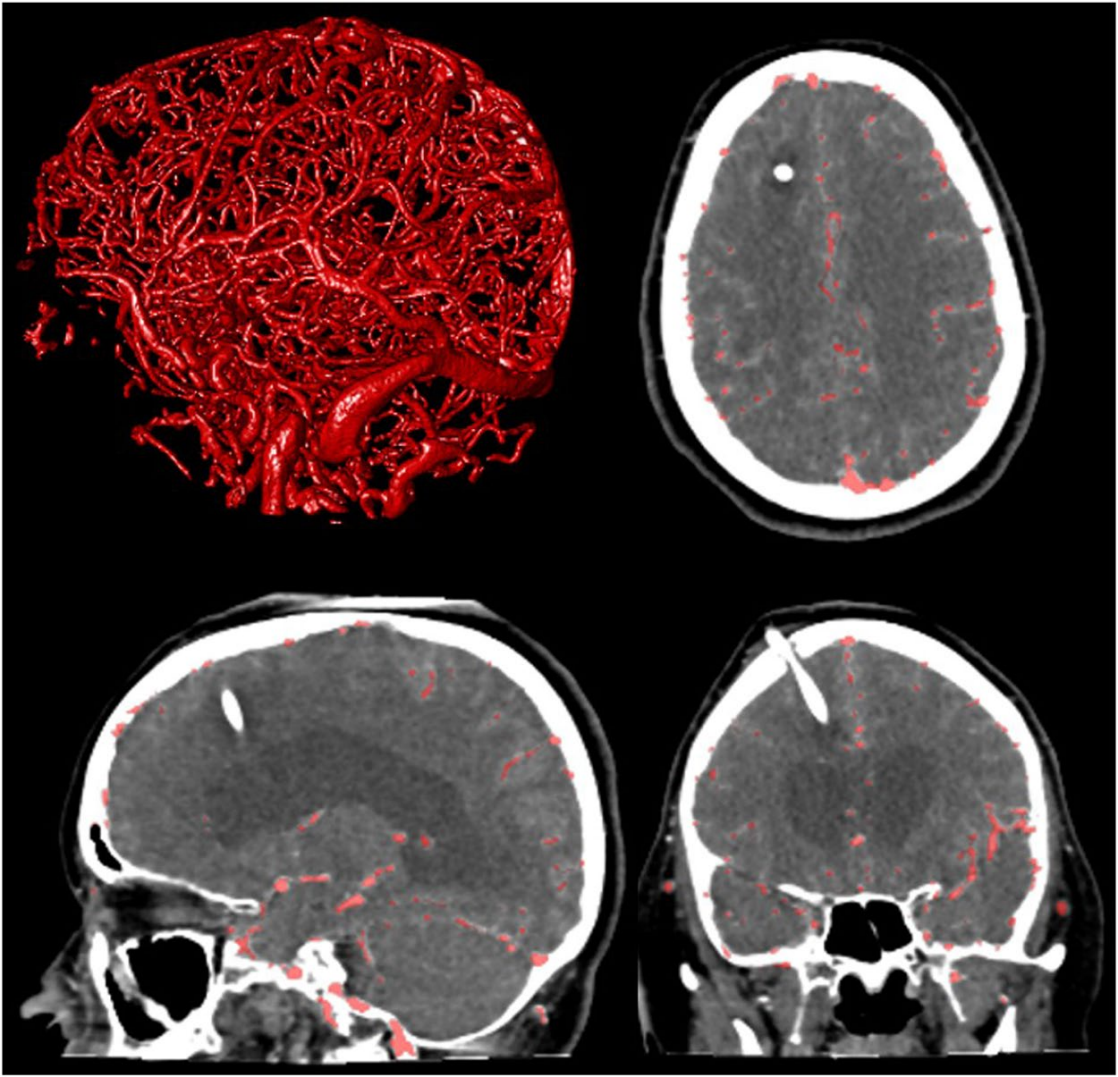
Axial, coronal, sagittal view and 3D rendering (clock-wise, from top-right) in a patient with a ventricular shunt. Overlay in red depicting the vessel segmentation results.

**Figure 7:**
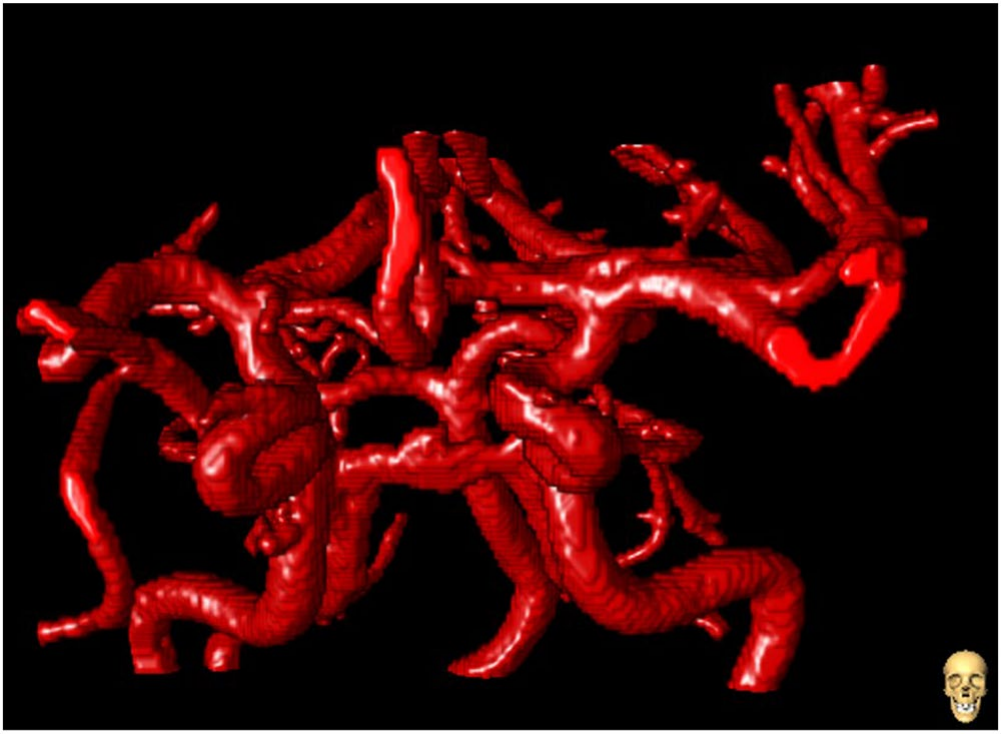
Anterior-Posterior view of a 3D rendering vessel segmentation of the circle of Willis.

The vast majority of the vessel segmentations were evaluated as excellent (Fig. 5). Presence of foreign objects or severe trauma proved no difficulty for the method nor resulted in over segmentation. Figure 6 shows a patient with foreign objects implanted as a result of surgical intervention. The skull base region is known to be the most difficult region for vessel segmentation. This region holds the ICA and a network of small veins intertwined surrounding the ICA, making distinguishment difficult. This region of interest in cerebral vessel segmentation was segmented in detail (Fig. 7).

### Quantitative Evaluation

The segmentations were compared with the reference standard provided by the manual annotations for quantitative evaluation. The same metrics as described in Section *Observer Variability* have been used. The results of the quantitative evaluation for 143 4D CT images are summarized in Table 3. The overall results and results per subvolume are presented. Subvolume 1 included the ICA, the CoW and the middle cerebral artery (MCA), subvolume 2 was defined in the frontal area including the anterior cerebral artery (ACA), subvolume 3 and 4 were defined left and right in the brain including the M2 and M3 vessel segments, and finally subvolume 5 at the parieto-occipital included the transverse sinus and the superior sagittal sinus.

**Table 3:** Quantitative evaluation of the segmentation results overall (n = 143) and per subvolume, reported as mean ± standard deviation. DSC is Dice similarity coefficient, HD is Hausdorff distance, MHD is modified HD, 95% HD is 95^th^ percentile HD, CMD is contour mean distance and AVD is absolute volume difference.

	Overall (n = 143)	Subvolume 1 (n = 23)	Subvolume 2 (n = 29)	Subvolume 3 (n = 33)	Subvolume 4 (n = 33)	Subvolume 5 (n = 25)
DSC	0.91 ± 0.07	0.91 ± 0.06	0.91 ± 0.08	0.90 ± 0.08	0.92 ± 0.07	0.93 ± 0.07
HD (mm)	8.05 ± 3.46	8.74 ± 3.20	7.64 ± 2.82	7.20 ± 2.56	7.14 ± 2.81	10.20 ± 4.85
MHD (mm)	0.23 ± 0.22	0.30 ± 0.25	0.22 ± 0.21	0.26 ± 0.24	0.18 ± 0.16	0.20 ± 0.22
95% HD (mm)	1.32 ± 1.67	2.14 ± 2.06	1.33 ± 1.76	1.49 ± 1.73	0.93 ± 1.16	0.82 ± 1.27
CMD (mm)	0.26 ± 0.24	0.37 ± 0.30	0.23 ± 0.21	0.27 ± 0.24	0.20 ± 0.17	0.28 ± 0.27
AVD (%)	14.17 ± 18.90	11.51 ± 13.80	14.96 ± 22.26	19.48 ± 23.06	11.83 ± 13.53	11.76 ± 17.11
Sensitivity	0.938	0.931	0.946	0.941	0.897	0.966
Specificity	0.997	0.995	0.998	0.998	0.998	0.997
Accuracy	0.995	0.992	0.997	0.996	0.994	0.996

**Figure 8:**
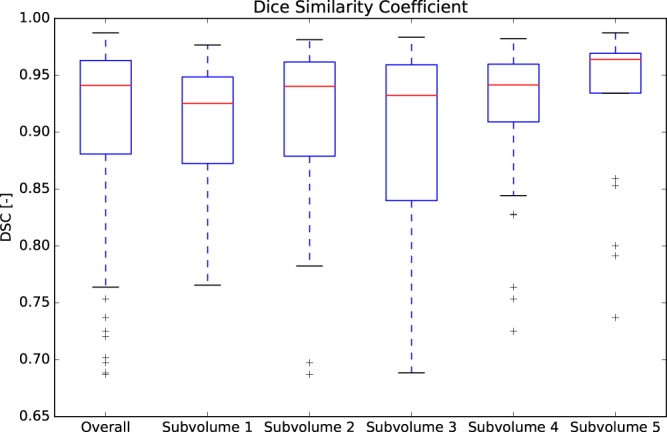
Boxplot of the DSC. The overall results are represented by the first bar (n = 143). The results per subvolume are shown in the remaining bars.

**Figure 9:**
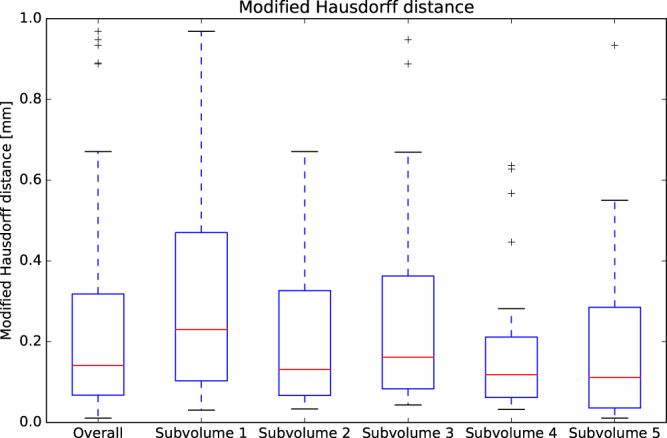
Boxplot of the MHD. The overall results are represented by the first bar (n = 143). The results per subvolume are shown in the remaining bars.

A boxplot graphical summary of the DSC score and the MHD is shown in Figs 8 and 9 respectively. Overall, the sensitivity, specificity, and accuracy of cerebral vasculature segmentation reported for all 143 patients were 0.938, 0.997 and 0.995 respectively.

## Discussion

In this work we have presented a method for full cerebral vessel segmentation in 4D CT using a pattern recognition framework with minimal pre- and postprocessing. The results show that the method correctly handles foreign objects without losing segmentation accuracy. Furthermore, our method is able to segment vessels of different sizes at different locations.

The presented method requires no tracking or active contours as these may be unable to handle the discontinuities in the vessels due to steno-occlusive disease, vessel wall pathology, calcification, or artifacts resulting from foreign objects. In addition, natural intensity differences throughout the vascular system in the brain may pose problems for these approaches. Intensity variations limit the application of global parameters and may require extra user input if the method depends on a manual chosen starting point.

The novelty of our method lies in two distinct features. First, the WTV image, which represents the intensity variance in temporal direction. This feature reduces the dimensionality of the image data and is highly sensitive to contrast variation over time. It is therefore very suitable for the visualization and detection of vessels in 4D CT. In addition, in contrast to approximated measures as the area under the curve, the WTV is parameter free and independent of the temporal distance between the volumetric acquisitions of the 4D CT protocol. The second feature is the use of the parameters (mean, standard deviation, mode and entropy) of a local intensity histogram to train the classifier. By analyzing the contrast variation in a histogram in a subvolume, each vessel, regardless of size, can be detected by the profile represented in the parameters of the histogram. The local histogram features are preferable to global thresholds as smaller vessel segments may be under segmented or missed as they fall below the global threshold dominated by the large vessels. By comparing vessel intensity to its immediate surrounding this can be avoided, as even the smallest vessels containing contrast agent have a higher intensity than the environment.

The method used pre- and postprocessing steps. The candidate vessel selection preprocessing step prior to the classification removes voxels which do not show any contrast variation over time. Removing these voxels reduces the computation time without losing possible vessel voxels.

One postprocessing step removed small objects from the segmentation result. The component size constraint of 25 voxels translates to 2.3 *μ*L. These small objects mainly occurred outside the cranial cavity around the facial area of the patient, due to noise, or eye or jaw movement during the acquisition.

Another postprocessing step was applied to compensate for under segmentations in large vessels. Under segmentation can occur in the main supplying arteries and main draining veins (e.g. the ICA and the transversus sinus). A reason for this under segmentation is a high blood flow causing high contrast variations over time. These high flows may not be captured by the 19 patients in the training set, resulting in holes in the segmentation. Although the classifier was trained with only 19 partially annotated patients, the method already performed well with this limited representation of the full population. Extra training sets can be added from patients with various arterial and venous flow speeds to capture a wider range in contrast variation to further increase the robustness of the method.

The inter-observer variability demonstrates the difficulty in the definition of the vessel border. The low inter-observer variability DSC of 0.75 ± 0.06 can be explained by the small size of the distal intracranial vessels. A vessel in this region can amount to merely two or three voxels in diameter. With a low component size the DSC is then easily affected. A 95% HD of 7.57 ± 3.07 mm is reported, which translates to a few voxels on both sides of the vessel, indicating that there is discrepancy on the definition of the exact border of the vessel. An exact definition of the vessel boundary is difficult because of the partial volume voxels. Also, what appears as vessel boundaries in the image is influenced by other factors including the injection rate of the contrast agent, the CT acquisition protocol, cardiovascular condition of the patient, and window width and level settings.

The proposed method has proven to be robust by evaluating on a large database comprised of 242 patients with foreign objects and image artifacts found in everyday clinical practice. The failures found were a result of insufficient intra-patient registration or as a result of acquisition artifacts resulting in loss of diagnostic value. Image artifacts caused by surgical intervention (e.g. clip, coil or drain) posed no problem for the method and did not result in under or over segmentation. Only severe movement between temporal acquisitions that has not been mitigated by the intra-patient registration showed to be a problem for the method. Smaller movement outside the cranial cavity only proved to be a problem in a small set of patients. The intra-patient registration registers bone tissue in each time point to the first time point. Soft tissues as the eyes and nose are not registered and can cause motion artifacts after the registration. Although our method handles minor soft tissue movements without any problems, severe movement of the jaw and nose may result in over segmentation.

Accurate and complete vessel segmentation in different brain regions is shown by the results in Table 3. The different subvolumes contained vessels of varying sizes, both arterial and venous. The DSC score showed similar results regardless of different subvolume evaluation region, which further supports the claim of independence towards vessel size or location. The MHD showed a distance similar to or smaller than the voxel spacing of 0.43 mm for all subvolumes combined as well for individual subvolumes, indicating a segmentation accurate to or below a single voxel. The method was not explicitly evaluated for topological consistencies, instead, we used similar evaluation measures as were used in some of the larger vessel segmentation challenges^18–20^.

Temporal information, in combination with contrast agent, is important for vessel segmentation as is reflected by the WTV feature. The added value of 4D CT with improved evaluation of intracranial hemodynamics comes at a cost, as a 4D CT protocol is associated with a higher radiation dose. Although 4D CT imaging is not common practice, applications of 4D CT are expanding^21^. We expect 4D CT to become a single acquisition for stroke workup as it contains both noncontrast CT and CTA information^22–24^. These modalities might be reconstructed from a 4D CT acquisition, resulting in a reduction of acquisitions and radiation dose. In addition, studies suggest that 4D CT can be acquired at half the dose of standard clinical protocol^25^, further reducing the radiation dose for the patient.

Although the images for this study were acquired with a high-end 320-row detector scanner, the voxel based classification method is not constrained to a specific image size nor images from scanners with a different number of detectors.

To the best of our knowledge this is the largest quantitative and qualitative evaluation reported for vessel segmentation in general. Robust cerebral vessel segmentation in 4D CT has not been performed on a similar scale.

In conclusion, we presented a method using candidate selection combined with a RF classifier trained with localized histogram features to segment a full cerebral vasculature. The method provides accurate and robust vessel segmentation on large amounts of everyday clinical data and forms the cornerstone of many subsequent applications.

## Methods

We propose a three step approach: first, coarse candidate vessel detection, then application of a random forest (RF) classifier^26^ using local and global image features and finally postprocessing to ensure full and smooth connectivity. The RF classifier is a supervised learning method that enables the learning of the background and the properties of vessels with different sizes and intensities. In addition, RF enables the learning of foreign objects or image artifacts which may be present in the data through a number of decision trees. The method was developed using the Insight Segmentation and Registration (ITK)^17^ library.

### Candidate Selection

The candidate vessel voxels were selected from the temporal variance image weighted to the exposures of the individual time points to maximize the signal to noise ratio. This WTV image is sensitive to contrast variation and is described in Section *Image Features*. A threshold of *μ* + 1.5*σ* was applied, with mean, *μ* and standard deviation, *σ* calculated from the intensity histogram of the WTV.

### Image Features

The image features used to train the classifier were the Weighted Temporal Average (WTA), WTV, local histogram features, the distance to the intracranial cavity, the eigenvalues of the Hessian system and *T*_0_. Each of these features will be discussed in the following sections.

#### Weighted Temporal Average

The first image feature is the temporal average image. The image noise differs per time point, because the volumetric acquisitions are acquired with different exposures settings. To achieve the highest signal to noise ratio, assuming that quantum noise is the dominating source of image noise in CT, which is the case for acquisition protocols used in standard clinical practice, the individual time points were weighted according to their individual exposure (*E*_*i*_). The resulting feature is called the Weighted Temporal Average^27^:

1 $${{\rm{WTA}}}_{x,y,z}=\sum _{i=0}^{T}{\omega }_{i}\cdot {I}_{x,y,z,i}$$

with the weight factor of each time point given by ${\omega }_{i}=\tfrac{{E}_{i}}{{\sum }_{i}{E}_{i}}$ with ${\sum }_{i}{E}_{i}$ the sum of all exposures over all time points.

#### Weighted Temporal Variance

**Figure 10:**
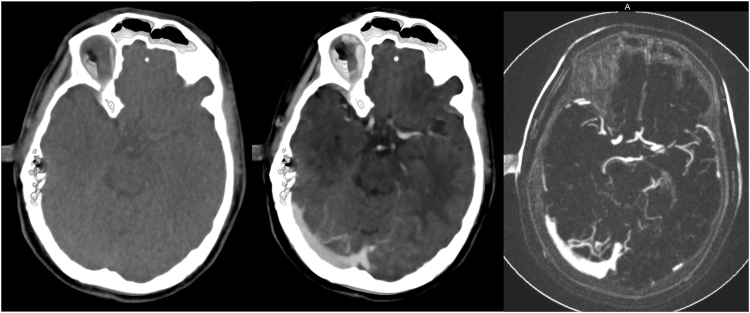
Left to right: First time point of 4D CT acquisition, WTA, and WTV. The WTV is highly sensitive to contrast changes over time making it a promising feature for vessel segmentation.

Similar as to the WTA, the temporal variance can be calculated with a weighted quadratic difference to the WTA. The WTV holds the largest contrast between tissue containing contrast agent and background tissue (Fig. 10) and is given by:

2 $${{\rm{WTV}}}_{x,y,z}=\sqrt{\sum _{i=0}^{T}{\omega }_{i}\cdot {({{\rm{WTA}}}_{x,y,z}-{I}_{x,y,z,i})}^{2}}$$

#### Local Histogram Features

To capture local image properties such as varying vessel intensity values, an intensity histogram is computed within a 5 × 5 × 5 and 9 × 9 × 9 neighborhood in the WTV image. The following histogram parameters were included: the mean, standard deviation, mode and the entropy given by $E=-{\sum }_{i}{p}_{i}\cdot {log}_{2}({p}_{i}+\varepsilon )$, with *p*_*i*_ the probability of intensity *i*, and *ε* = 0.0001 to prevent zero-logarithm calculation.

#### Distance to Intracranial Cavity

An intracranial cavity mask is calculated and used as distance feature. The cranial cavity contains all soft tissues and cerebrospinal fluid, including the meninges, cerebrum and ventricles, cerebellum and brain stem. The cavity mask was created by a multi-atlas registration with label fusion followed by a geodesic active contour levelset refinement of the segmentation^28^. The internal carotid arteries and jugular veins are located outside of the intracranial cavity mask. These will be missed if the cavity mask is added as a binary feature, therefore the Euclidean distance to the border of the intracranial cavity mask was computed. Distances from inside the mask to the border were denoted as negative distances, distances from outside the mask to the border were denoted as positive distances.

#### Hessian

The eigenvalues of the Hessian system are calculated on the WTV at four different scales on an evenly distributed range from 0.5 to 2.0 mm.

#### T_0_

The 4D CT acquisition contains 19 volumetric acquisitions. The first time point volume was selected to represent the tissue with minimal contrast. This volume was used in the feature set as *T*_0_.

### Classifier

An RF classifier is applied to the candidate vessel voxels. The RF classifier was trained with 100 trees and a maximum tree depth of 30.

### Postprocessing

After the RF classification two postprocessing steps were applied. First, a connected components (26-neighborhood) analysis was carried out, discarding components smaller than 25 voxels to remove background noise. Second, morphological hole filling with a 3 × 3 × 3 kernel size was carried out for 10 iterations to fill the center of some larger vessels such as the ICA in the event of under segmentation by the classifier.

## Materials

### Patient Data

This retrospective study was approved by our local institutional review board and the requirement for informed consent was waived. In total, 264 consecutive patients (age: 68*y* ± 15.0, male: 149 (55.6%)) with suspicion on acute ischemic stroke who were admitted to the emergency department of our academic hospital, between January 1st 2014 and December 31st 2015, were retrospectively collected for this study. Exclusion criteria were severe motion artifacts resulting in loss of diagnostic value (n = 3). This was determined from the radiology report provided by the radiologist on call at time of admission. Image acquisition was done on a 320-row Toshiba Aquilion ONE CT scanner (Toshiba Medical Systems Corporation, Japan). The protocol consisted of 19 whole brain volume acquisitions starting with a single high dose acquisition at 200 mAs 5 seconds after contrast injection, followed by 13 scans every 2 seconds at 100 mAs, followed by 5 scans every 5 seconds at 75 mAs. Each volumetric acquisition was made at 80 kV at 0.5 seconds rotation time with 16 cm z-coverage. A dose of 80 ml Iomeron is injected in the antecubital vein at the start of the first acquisition. Image reconstruction was done with a smooth reconstruction kernel (FC41), resulting in image sizes of 512 × 512 × 320 voxels with voxel sizes of 0.43 × 0.43 × 0.5 mm. All temporal volumes were rigidly registered to the first time point to resolve any patient movement during the acquisition.

### Volumetric Cluster Annotation and Segmentation Tool (VCAST)

Annotating vessels in 3D is a laborious and difficult task because of their small sizes and the presence of partial volume voxels, and because vessels meander through the different orthogonal planes. To facilitate the annotation process, a dedicated 3D annotation tool called volumetric cluster annotation tool was developed^29^. VCAST supports interactive region growing and super voxel grids overlaid on the input image to let the user assign or reject vessel labels to clusters of voxels at multiple scales. Additional functionalities include the ability to define subregions confining the region growing algorithm, a 3D rendering of the topological information of the annotations and a colour map defining connected regions with the same colour. Annotations were done on the WTA because of its maximal signal to noise ratio and on the WTV because of its high sensitivity to contrast voxels. Observers were able to switch between these reconstructions for optimal interaction.

### Reference Standard

**Figure 11:**
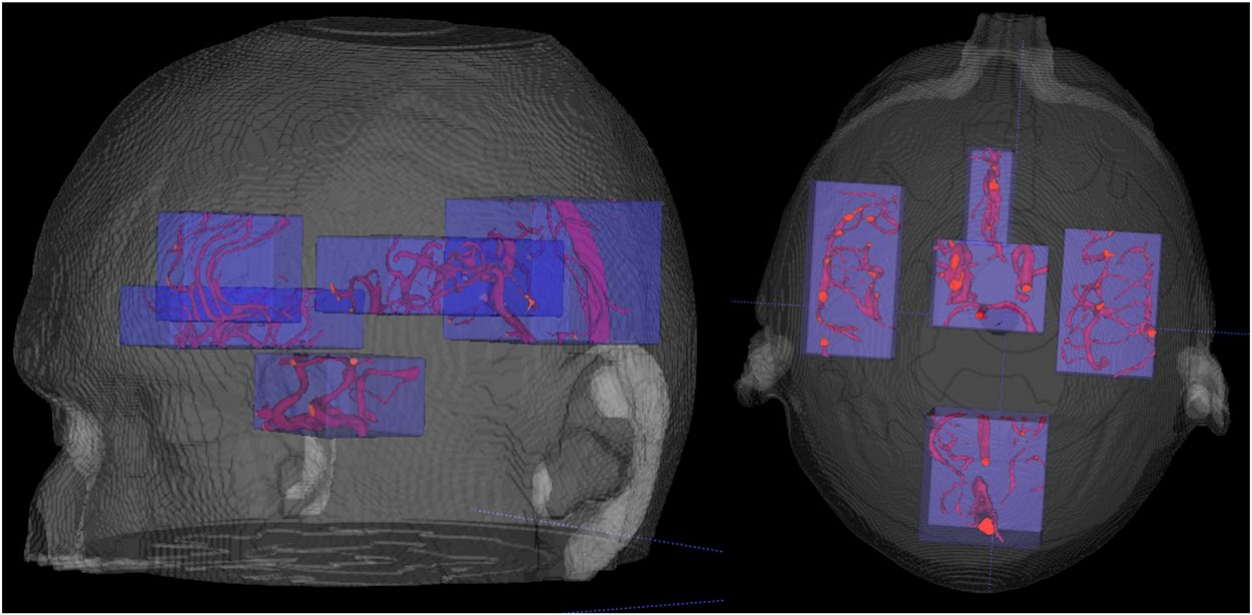
3D rendering of approximate locations of the five subvolumes. The subvolumes cover the MCA, CoW and parts of the distal and posterior circulation.

Annotating the full cerebral vasculature is too labour intensive and not required, because many vessel segments have similar appearances and intensities. Therefore, annotations were carried out in five subvolumes representing different parts of the vasculature, as shown in Fig. 11.

Observers were asked to annotate high confidence vessel voxels in these subvolumes, thereby focusing more on the completeness of the vasculature and less on the accuracy of the vessel boundaries at a single voxel level. In the training dataset (n = 19) all five subvolumes were annotated in each patient. In the testing dataset (n = 159) one of the five subvolumes was randomly selected per patient for annotation. These annotations were carried out by two medical assistants, supervised by an experienced neuroradiologist (FJAM).

### Evaluation Measures

The segmentations were compared with the reference standard provided by the annotations using the following measures: sensitivity, specificity, accuracy, DSC, HD, MHD, 95% HD, CMD, and AVD. The MHD is defined as the maximum value of the mean distance calculated between two objects. The CMD is defined as the mean distance between the segmentations along their boundary voxels. The sensitivity, specificity, and accuracy were calculated within the annotated subvolume on a voxel basis. The mean and standard deviation was taken over all patients for the other measurements. These evaluation measures are similar to the measures used in some vessel segmentation challenges^18–20^.

The goal of our method is to robustly segment a complete cerebral vasculature. To that end, the focus of the evaluation will not be around the border of vessel lumen. Therefore, the lumen boundaries are excluded in the final evaluation by dilating the manual annotations by 1 voxel (3 × 3 × 3 kernel). Applying this boundary mask will only boost the specificity of the algorithm in regions where exact definitions of the vessels border is difficult, but it will not affect the sensitivity.

## Experiments

**Figure 12:**
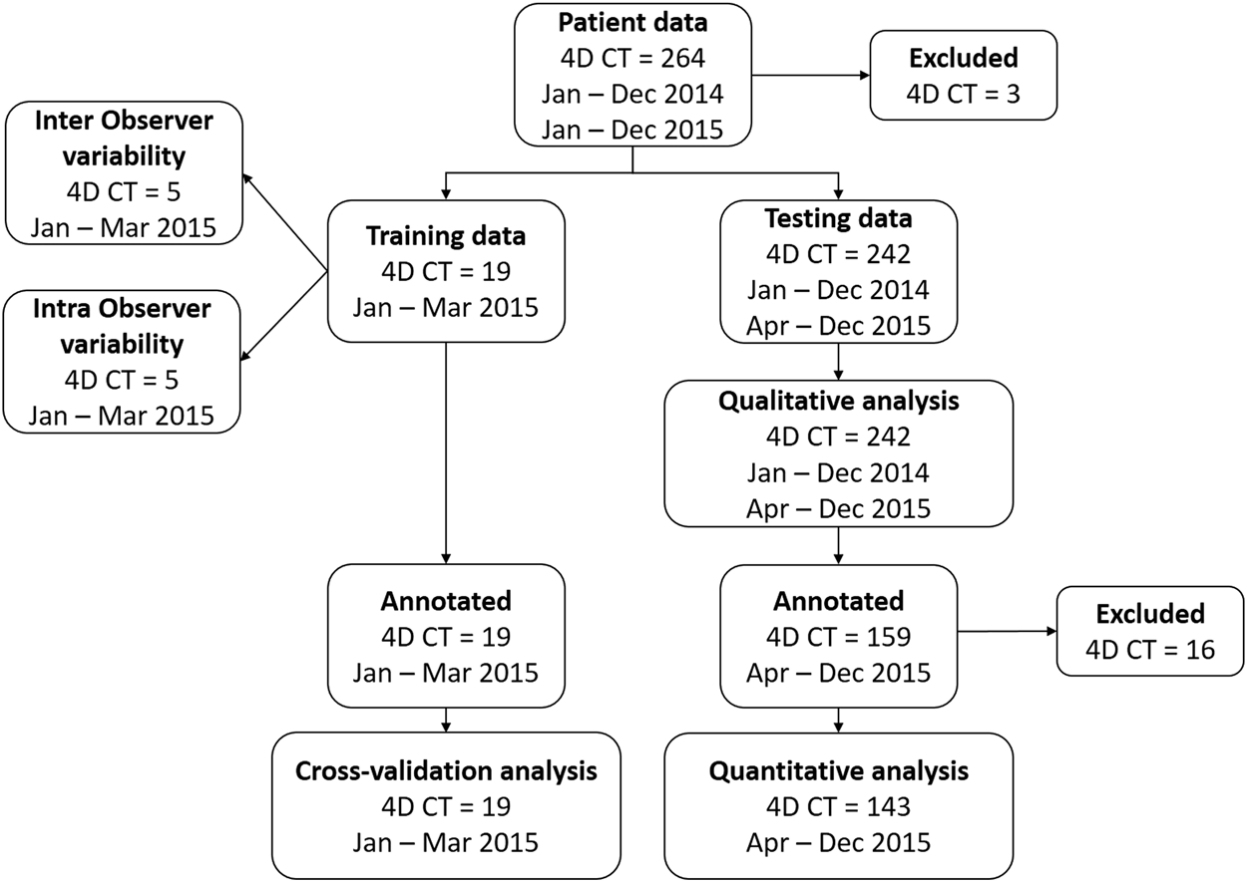
Schematic overview of study data.

A schematic overview of the acquired imaging data and the experiments described below is shown in Fig. 12.

### Training and Validation

The annotation of the training set provided positive samples from 5 subvolumes within 19 patients. A random subset of 10% of the annotated vessel voxels was used as positive samples to train the classifier. A dataset of the same size as the positive data set was randomly sampled from all nonannotated voxels within the subvolumes. These voxels served as background. The RF classifier was optimized using leave-one-out crossvalidation within the training set. The accuracy of the receiver operator characteristics (ROC) analysis and visual inspection of the output was used to determine the optimal operating point.

### Observer Variability

A subset of 5 patients randomly selected from the training set were annotated by both trained observers. The same subset was annotated by one observer once again after a waiting period of two weeks. All evaluation measures described in Section *Evaluation Measures* were reported for the inter- and intra-observer variability. Quantitative evaluation of observer variability was performed on the full annotation without excluding boundary voxels. Because the annotation sets consisted of five nonconnected subvolumes the distance evaluation measures were first calculated per subvolume, then combined between subvolumes according to their measurement. That is, the mean for CMD and the maximum for HD, MHD, and for 95% HD. Finally, the mean and standard deviation per evaluation measure were taken over all patients and reported.

### Qualitative Evaluation

The robustness was verified by imposing minimal exclusion criteria on the test data set, thus resembling clinical practice. All vessel segmentations were initially inspected to assess failures. Failure was reported in cases of severe under or over segmentation. In addition, the entire branch network of the cerebral structure was investigated for completeness.

### Quantitative Evaluation

The completeness of vasculature was assessed by quantitatively evaluating different parts of the cerebral structure in a large number of patients. The quantitative evaluation was performed on one of five randomly annotated subvolumes. These subvolumes were defined in a similar region as with the training data, described in Section *Results* under *Quantitative Evaluation*.

## References

[1] Powers WJ (2015). 2015 American Heart Association/American Stroke Association focused update of the 2013 guidelines for the early management of patients with acute ischemic stroke regarding endovascular treatment. A guideline for healthcare professionals from the American Heart Association/American Stroke Association. Stroke.

[2] Kirbas C, Quek F (2004). A review of vessel extraction techniques and algorithms. ACM Comput. Surv. (CSUR).

[3] Lesage D, Angelini ED, Bloch I, Funka-Lea G (2009). A review of 3D vessel lumen segmentation techniques: models, features and extraction schemes. Med. Image Analysis.

[4] Flasque N, Desvignes M, Constans JM, Revenu M (2001). Acquisition, segmentation and tracking of the cerebral vascular tree on 3D magnetic resonance angiography images. Med. Image Analysis.

[5] Molyneux AJ (2005). International subarachnoid aneurysm trial (ISAT) of neurosurgical clipping versus endovascular coiling in 2143 patients with ruptured intracranial aneurysms. Lancet.

[6] Bouix S, Siddiqi K, Tannenbaum A (2005). Flux driven automatic centerline extraction. Med. Image Analysis.

[7] Larson DB, Johnson LW, Schnell BM, Salisbury SR, Forman HP (2011). National trends in CT use in the emergency department: 1995–2007 1. Radiol..

[8] Talwalkar, A. & Uddin, S. Trends in emergency department visits for ischemic stroke and transient ischemic attack: United States, 2001–2011. 2015 (US Department of Health and Human Services, Centers for Disease Control and Prevention, National Center for Health Statistics, 2015).

[9] Quaday KA, Salzman JG, Gordon BD (2014). Magnetic resonance imaging and computed tomography utilization trends in an academic emergency department. Am. J. of Emerg. Medicine.

[10] Mendrik A (2010). Automatic segmentation of intracranial arteries and veins in four-dimensional cerebral CT perfusion scans. Med. Phys..

[11] Robben D (2016). Simultaneous segmentation and anatomical labeling of the cerebral vasculature. Med. Image Analysis.

[12] Kiros, R., Popuri, K., Cobzas, D. & Jagersand, M. Stacked multiscale feature learning for domain independent medical image segmentation. In *Machine Learning in Medical Imaging*, 25–32 (Springer, 2014).

[13] Rudyanto RD (2014). Comparing algorithms for automated vessel segmentation in computed tomography scans of the lung: The VESSEL12 study. Med. Image Analysis.

[14] Dice LR (1945). Measures of the amount of ecologic association between species. Ecol..

[15] Huttenlocher DP, Klanderman G, Rucklidge WJ (1993). Comparing images using the Hausdorff distance. IEEE Transactions on Pattern Analysis and Mach. Intell..

[16] Dubuisson M-P, Jain AK (1994). A modified Hausdorff distance for object matching. In Proceedings of the 12th IAPR International Conference on Pattern Recognition.

[17] Yoo, T. S. *et al*. Engineering and algorithm design for an image processing api: a technical report on itk-the insight toolkit. *Studies in health technology and informatics* 86–592 (2002). 15458157

[18] Schaap M (2009). Standardized evaluation methodology and reference database for evaluating coronary artery centerline extraction algorithms. Med. Image Analysis.

[19] Hameeteman K (2011). Evaluation framework for carotid bifurcation lumen segmentation and stenosis grading. Med. Image Analysis.

[20] Kirisli HA (2013). Standardized evaluation framework for evaluating coronary artery stenosis detection, stenosis quantification and lumen segmentation algorithms in computed tomography angiography. Med. Image Analysis.

[21] Kortman HGJ (2015). 4D-CTA in neurovascular disease: A review. Am. J. of Neuroradiol..

[22] Smit EJ (2013). Timing-invariant imaging of collateral vessels in acute ischemic stroke. Stroke.

[23] Oei, M. T. H. *et al*. Interleaving cerebral CT perfusion with neck CT angiography. Part I: Proof of concept and accuracy of cerebral perfusion values. *Eur. Radiol*. **27**, 2649–2656 (2017). 10.1007/s00330-016-4577-y PMC5409805 27718078

[24] Oei, M. T. H. *et al*. Interleaving cerebral CT perfusion with neck CT angiography. Part II: Clinical implementation and image quality. *Eur. Radiol*. **27**, 2411–2418 (2017). 10.1007/s00330-016-4592-z PMC5408041 27651144

[25] Manniesing R, Oei MTH, van Ginneken B, Prokop M (2016). Quantitative dose dependency analysis of whole-brain CT perfusion imaging. Radiol..

[26] Breiman L (2001). Random forests. Mach. Learn..

[27] Manniesing, R. *et al*. White matter and gray matter segmentation in 4D computed tomography. *Nat. Sci. Reports* **7** (2017). 10.1038/s41598-017-00239-z PMC5428067 28273920

[28] Patel A (2017). Robust cranial cavity segmentation in ct and ct perfusion images of trauma and suspected stroke patients. Med. Image Analysis.

[29] van de Leemput, S., Meijer, F. J. A., Prokop, M. & Manniesing, R. Cerebral white matter, gray matter and cerebrospinal fluid segmentation in CT using VCAST: a volumetric cluster annotation and segmentation tool. In *European Congress of Radiology* (2017).

[30] Passat N, Ronse C, Baruthio J, Armspach J-P, Maillot C (2006). Magnetic resonance angiography: From anatomical knowledge modeling to vessel segmentation. Med. Image Analysis.

[31] Passat N (2005). Region-growing segmentation of brain vessels: An atlas-based automatic approach. J. of Magn. Reson. Imaging.

[32] Hassouna MS, Farag A, Hushek S, Moriarty T (2006). Cerebrovascular segmentation from TOF using stochastic models. Med. Image Analysis.

[33] Yan P, Kassim AA (2006). Segmentation of volumetric MRA images by using capillary active contour. Med. Image Analysis.

[34] Lorigo LM (2001). Curves: Curve evolution for vessel segmentation. Med. Image Analysis.

[35] Law MWK, Chung ACS (2007). Weighted local variance-based edge detection and its application to vascular segmentation in magnetic resonance angiography. IEEE Transactions on Med. Imaging.

[36] Gao X (2010). A fast and fully automatic method for cerebrovascular segmentation on time-of-flight (TOF) MRA image. J. of Digit. Imaging.

[37] Wang R (2015). Threshold segmentation algorithm for automatic extraction of cerebral vessels from brain magnetic resonance angiography images. J. of Neurosci. Methods.

[38] Bogunovic H, Pozo JM, Cardenes R, Roman LS, Frangi AF (2013). Anatomical labeling of the circle of willis using maximum a posteriori probability estimation. IEEE Transactions on Med. Imaging.

[39] Manniesing R (2006). Level set based cerebral vasculature segmentation and diameter quantification in CT angiography. Med. Image Analysis.

[40] Schaap M (2007). Bayesian tracking of tubular structures and its application to carotid arteries in CTA. In Medical Image Computing and Computer-Assisted Intervention.

[41] Hernandez M, Frangi A (2007). Non-parametric geodesic active regions: Method and evaluation for cerebral aneurysms segmentation in 3DRA and CTA. Med. Image Analysis.

